# Optimized fiducial marker placement using B‐spline surface modeling and graph theory for Cyberknife stereotactic body radiotherapy for superficial tumors

**DOI:** 10.1002/pro6.70017

**Published:** 2025-06-19

**Authors:** Jing Huang, Xianlong Xiong, Cheng Chen, Yuhan Li, Ruijie Wang, Zhitao Dai

**Affiliations:** ^1^ School of Computer Science and Artificial Intelligence Wuhan University of Technology Wuhan China; ^2^ Department of Radiation and Medical Oncology Zhongnan Hospital of Wuhan University Wuhan Hubei China; ^3^ National Cancer Center/National Clinical Research Center for Cancer/Cancer Hospital & Shenzhen Hospital Chinese Academy, of Medical Sciences and Peking Union Medical College Shenzhen China

**Keywords:** Fiducial Marker Placement, CyberKnife, Stereotactic Body Radiotherapy, Superficial Tumor

## Abstract

CyberKnife, an established noninvasive stereotactic radiotherapy technology, has been extensively utilized to treat various malignancies because of its high precision and conformal dose delivery. The success of CyberKnife treatment is crucially dependent on optimal fiducial marker placement. This study introduces a novel fiducial marker placement planning algorithm tailored for superficial tumors, which are located 20–50 mm beneath the epidermis. A retrospective analysis was performed on the data collected from three patients with thymus, breast, and submandibular gland tumors. This algorithm generated potential implantation sites by constructing and optimizing a B‐spline surface around the tumor. Candidate points were filtered using multi‐criteria constraints: (1) a minimum of 18‐mm inter‐marker distance, (2) angular separation >30°, and (3) nonoverlapping visibility in 45° oblique digital reconstructed radiographs. To enhance the computational efficiency, a kd‐tree spatial indexing structure was integrated with graph theory, specifically the Bron–Kerbosch algorithm for maximal clique detection. The proposed method achieved a time complexity of $O(\mathrm{mlogm}+m^{2}+3^{\frac{n}{3}})$, demonstrating a significant improvement over the brute‐force $O(n^{3})$ approach. The experimental results showed that our algorithm could efficiently plan fiducial marker placement, thereby simplifying the planning process and providing valuable technical support for CyberKnife treatments.

## Graphical abstract

This study introduces a Fiducial marker placement planning algorithm, specifically for superficial tumors that are located 20‐50 mm beneath the epidermis. The algorithm proposes numerous potential implantation sites by constructing and optimizing a B‐spline surface. The time complexity of the algorithm proposed in this study is O(mlogm+m^2^+3^(n/3)^), significantly faster than the brute‐force O(n^3^) approach. Experimental outcomes show that our algorithm can efficiently plan Fiducial marker placements, simplifying the planning process and providing valuable technical support for CyberKnife treatments.

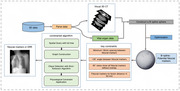

## 1 INTRODUCTION

Stereotactic body radiotherapy (SBRT) represents a revolutionary advancement in delivering precise radiation doses to tumors while sparing adjacent healthy tissues.^1^, ^2^ Two critical challenges in SBRT implementation are accurate tumor delineation and the mitigation of tumor motion.^1^ The CyberKnife system, which is renowned for its noninvasive stereotactic radiotherapy capabilities,^3^, ^4^, ^5^, ^6^ has gained widespread clinical adoption because of its high targeting precision, conformal dose distribution, and minimal treatment‐related toxicity. A key innovation of the CyberKnife technology is its real‐time tumor tracking system, which significantly enhances the irradiation accuracy for dynamically moving tumors, such as hepatic lesions.^7^, ^8^, ^9^, ^10^, ^11^, ^12^, ^13^, ^14^

**FIGURE 1:**
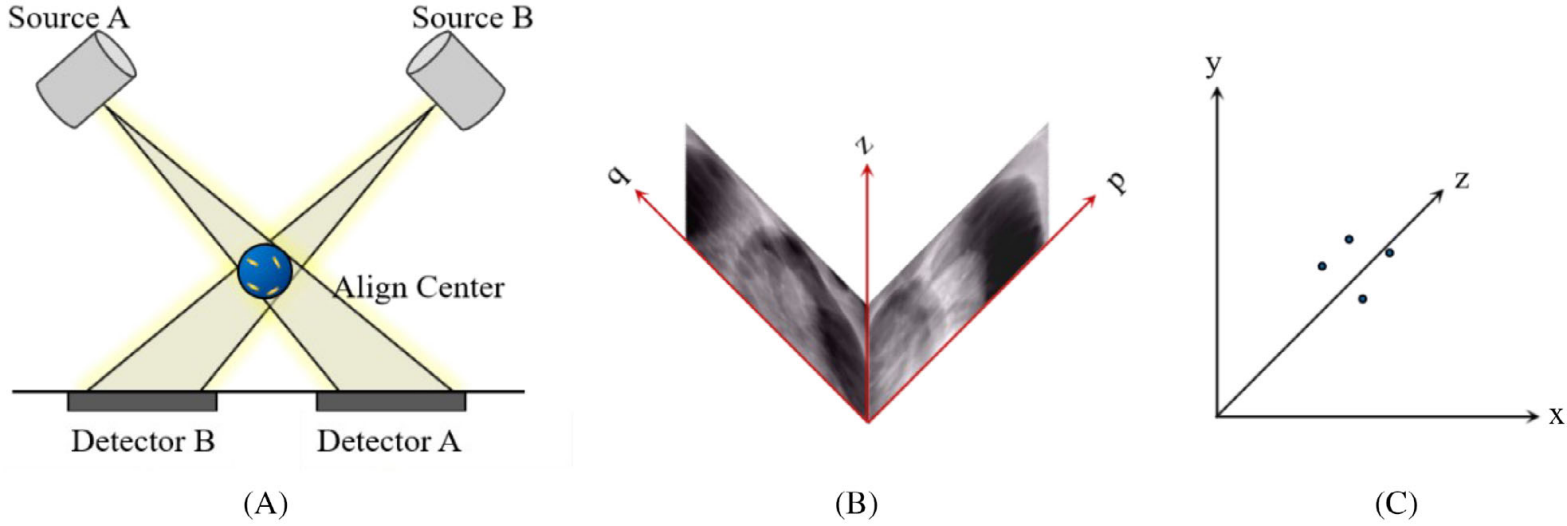
Diagram of the CyberKnife imaging system (A) and a schematic of coordinate transformation between the (B) image coordinate system (p, q, z) and (C) patient coordinate system (x, y, z). The blue sphere in panel (A) is the align center and the yellow bars shown inside it are fiducial markers. The p‐z and q‐z planes in panel (B) represent kV images constructed from detectors A and B, respectively. The small dots in panel (C) indicate fiducial positions in the patient coordinate system.

The CyberKnife system employs two ceiling‐mounted kilovoltage (kV) X‐ray sources paired with floor‐level detectors configured at orthogonal 45° angles to enable stereoscopic imaging, as shown in Figure 1(A). This geometry allows the three‐dimensional (3D) reconstruction of fiducial markers through the triangulation of radiographic projections. However, some tumors are difficult to visualize on X‐ray images because of the low contrast between them and the surrounding soft tissues. An effective solution to this problem is the use of implanted fiducial markers as surrogates for tumors. Fiducial markers, typically made from radiopaque materials, can be viewed from two orthogonal camera positions and their 3D coordinates can be reconstructed at the intersection of the back projections towards the source.^15^ The coordinates of the fiducials in the patient coordinate system (p,q,z) can be obtained from the coordinates in the image coordinate system (x,y,z), as shown in Figures 1(B) and (C). Details of the algorithm are available elsewhere.^16^, ^17^

Althoughm fiducial tracking theoretically requires at least one marker for translational motion correction, multiple markers (4–6 in clinical practice) are preferred to enable 6D motion tracking and improve error detection.^18^ The optimal marker placement must adhere to the following strict geometric constraints:

- ≥18‐mm inter‐marker distance
- >15 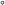 angular separation between marker pairs
- Nonoverlapping visibility in 45° oblique digital reconstructed radiographs (DRRs)
- ≤50 mm distance between the marker and tumor centroids

Current clinical practice relies heavily on manual marker placement guided by computed tomography (CT)/magnetic resonance imaging and physician experience.^19^, ^20^ However, this approach has inherent limitations: (1) lack of standardization owing to interoperator variability, (2) increased risk of critical organ injury near complex anatomies, and (3) time‐consuming iterative adjustments requiring multiple imaging sessions.

To address these challenges, we introduce a computer‐aided fiducial placement algorithm based on B‐spline surface modeling and constraint optimization. This method uses CT images of patients as its foundation and employs graphics techniques to construct B‐spline surfaces around the tumor,^21^, ^22^, ^23^, ^24^, ^25^ generating a plethora of potential fiducial marker placements and subsequently forming a complex set of fiducial marker points. Building on this, we applied the guiding principles for clinical fiducial marker placement to constrain and filter the fiducial marker point set, ensuring that all the collections of fiducial markers complied with the constraints. This provided a robust guarantee of the precision and safety of SBRT, thereby enhancing the tumor cure rate.

## 2 MATERIALS AND METHODS

**FIGURE 2:**
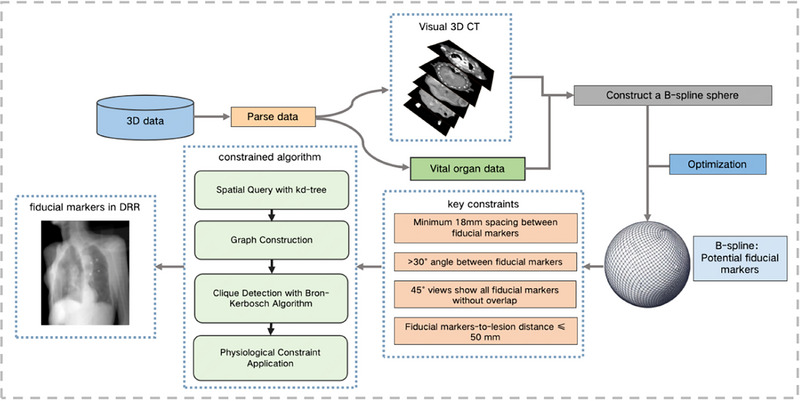
Flow chart of the process of precise fiducial marker placement for the treatment of superficial tumors using the CyberKnife technology.

The workflow of the proposed algorithm is illustrated in Figure 2. A 3D digital model of the tumor and organ tissues was constructed. B‐spline surfaces were used to fit a set of sampling points around the tumor lesion, each of which served as a potential location for fiducial marker placement. The number of candidate fiducial markers was initially defined as N, where N was ≥3. These candidate points were then screened based on their Euclidean distances and the internal angles of the triangles they formed to obtain a set of fiducial markers. Further constraints were applied to ensure that the projections of these fiducial markers did not overlap in the 45° oblique DRRs. This resulted in a final set of alternative fiducial markers.

### 2.1 Patient data collection and processing

Three patients who underwent CyberKnife treatment at our center were retrospectively selected for this study. The treatment sites in these three patients were the thymus, breast, and submandibular gland, with tumor depths of approximately 20–50 mm. CT simulations were performed using a GE Discovery RT 16‐slice CT scanner (slice thickness: 1.25 mm) with the patient in the supine position. The clinical target volume and critical structures were contoured jointly by an oncologist and radiologist based on the fusion of CT and magnetic resonance images or enhanced CT images on the Precision^TM^ system (Accuray Inc., Sunnyvale CA; version 1.1). The CT images and contour files were exported and processed using the PyDicom library. A comprehensive 3D visualization pipeline was implemented for analyzing tumor morphology and adjacent tissues, providing a foundation for fiducial planning.

### 2.2 B‐spline surface fitting

**FIGURE 3:**
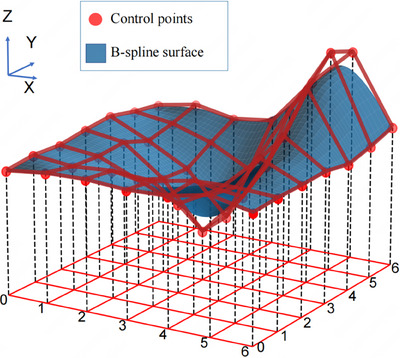
A schematic diagram of B‐spline surface fitting.

B‐spline surfaces are mathematical tools that are widely used in computer graphics and computer‐aided design/ manufacturing systems for modeling complex 3D surfaces. They are defined by the control points and knot vectors along the *u* and *v* directions, respectively. A schematic diagram of B‐spline surface fitting is shown in Figure 3. Each control point influences the surface morphology and placement, whereas the knot vectors govern the curvature and continuity of the curve. On a microscopic scale, a B‐spline surface can be perceived as the result of the summation of products between the base functions and control points. Macroscopically, the arrangement and distribution of the control points in space define the overall geometric attributes of the surface. Considering the exemplary performance of B‐spline surfaces in fitting complex 3D data, such as point cloud datasets, as well as their advantages in reducing the number of data points and subsequently diminishing computational and search complexities, B‐spline surfaces were chosen as the mathematical tool for constructing the tumor surface, as illustrated in Figure 3.

#### 2.2.1 B‐spline surface definition and control point optimization

In a 3D space, point datasets allow us to describe the surfaces of tumors. B‐spline surfaces can be defined by two sets of parameters: (1) the control points that dictate the shape of the surface and (2) the knot vectors that determine the curvature of the surface. In this study, the coordinates of the control points were obtained by sampling the point cloud data of the tumor surface.

To define a B‐spline surface S(u,v), let $P_{i,j}\;$ represent the control points, where i and j are the indices along the u and v directions, respectively. The surface is defined in Equation 1.

(1) $$S\;\left(u,v\right)=\sum_{i=0}^{m}\sum_{j=0}^{n}N_{i,p}\left(u\right)N_{j,q}\left(v\right)P_{i,j}$$

where $N_{i,p}\left(u\right)$ and $N_{j,q}\left(v\right)$ are the B‐spline basis functions defined along the u and v directions, respectively, and p and q are the orders of the B‐spline surface in the u and v directions, respectively.

To optimize the control points, we defined a least‐squares error function E, which is the sum of the squared Euclidean distances between the B‐spline surface and point cloud data representing the tumor surface. The computational expression is shown in Equation 2.

(2) $$E=\sum_{k=0}^{K-1}∥P_{k}-S\left(u_{k},v_{k}\right){∥}^{2}$$

$P_{k}$ represents a point in the point cloud, and $S(u_{k},v_{k})$ represents a point on the fitted surface at parameters $\left(u_{k},v_{k}\right)$. The optimized control points can be obtained by minimizing the error function E.

#### 2.2.2 Sample point generation

Once the B‐spline surface is constructed, sampling points can be generated on the surface by taking uniform values of the surface parameters (u,v). These sampling points represent potential precise location for placing fiducial markers on the tumor surface. In this step, it is necessary to ensure that the distance between the fiducial markers and tumor is less than the specified distance of 50 mm. The premise of using gold markers as surrogates for tumors is that the relative positions of the gold marker and tumor are fixed or change very little and their movements are synchronous. If the gold marker is too far from the tumor, the positions of the gold marker and tumor may change asynchronously, leading to a large tracking error. Given our study's focus on superficial tumors and in accordance with the definition of surface tumors, the sampling points created via the B‐spline surface were all less than 20–30 mm away from the tumor.

#### 2.2.3 Multirule constraint

We employ a series of 3D points to represent the locations of the fiducial markers, denoted as set G, where the location of each fiducial marker is represented as a 3D coordinate point $g_{i}=\left(x_{i},y_{i},z_{i}\right)$, and i is its index within set G. Firstly, at least three fiducial markers are required to establish stable spatial placement, i.e., N ≥3. We defined a series of medical constraints as mathematical constraints that describe the interrelationships among the fiducial markers. The first constraint is that the Euclidean distance between any pair of fiducial markers must be ≥18 mm, which is mathematically expressed in Equation 3.

(3) $$\begin{array}{c} ∥g_{i}-g_{j}∥=\sqrt{{\left(x_{i}-x_{j}\right)}^{2}+{\left(y_{i}-y_{j}\right)}^{2}+{\left(z_{i}-z_{j}\right)}^{2}}\;\geq 18\left(mm\right) \end{array}$$

Here, i ≠ j indicates any two fiducial markers. Next, we introduced an angular constraint. According to the CyberKnife manual, for any three arbitrarily selected distinct fiducial markers $g_{i}$, $g_{j}$, $g_{k}$ within set G, the angle between them must be >15°. However, the potential impact of factors such as fiducial marker displacement or organ movement after fiducial marker implantation may lead to changes in the relative positions of the fiducial markers. Therefore, we set the angle threshold to 30° in our optimization algorithm, resulting in a more robust fiducial marker geometry distribution.

(4) $$cos\theta =\frac{a\cdot b}{∥a∥∥b∥}$$

(5) $$cos^{-1}\left(cos\theta \right)\geq 30^{\circ }$$

The final constraint ensured that none of the fiducial markers overlapped in the two DRRs projected at a 45° angle to the human body. The locations of any two fiducial markers in 3D space are represented by the coordinates $g_{i}=\left(x_{i},y_{i},z_{i}\right)$ and $g_{j}=\left(x_{j},y_{j},z_{j}\right)$, respectively. To simulate the effect of 45° oblique irradiation, we defined the centroid of the tumor as the origin of the coordinate system. By applying a rotation matrix $R_{z}$ around the Z axis, we calculated the locations of the fiducial markers on the projection plane. The rotation matrix for a 45° rotation around the Z axis is given as:

(6) $$R_{z}=\left[\begin{array}{cc} \begin{array}{cc} \cos\left(45^{\circ }\right) & -\sin\left(45^{\circ }\right)\\ \sin\left(45^{\circ }\right) & \cos\left(45^{\circ }\right) \end{array} & \begin{array}{c} 0\\ 0 \end{array}\\ \begin{array}{cc} 0 & \;0 \end{array} & 1 \end{array}\right]\;$$

By multiplying the 3D coordinates for the location of each fiducial marker with the rotation matrix $R_{z}$, we obtained the new locations of the fiducial markers on the projection plane after a 45° rotation, denoted as $p_{i}$ and $p_{j}$ for $R_{z}\cdot g_{i}$ and $R_{z}\cdot g_{j}$, respectively. Here, $p_{i}$ and $p_{j}$ represent the projection coordinates of the fiducial markers $g_{i}$ and $g_{j}$ on the X‐Y plane after a 45° rotation. The clinical standard requires that $p_{i}$ and $p_{j}$ avoid overlapping on each projection plane rotated by 45°.

**ALGORITHM 1:** The fiducial marker placement planning algorithm.

**Require**: Center point, point array, tumor midpoint in Z axis	
**Ensure**: Fiducial markers (res)	
1:	res ← []
2:	Convert points to NumPy array
3:	Extract x, y, z coordinates
4:	Define Z axis threshold for filtering
5:	filtered_points ← Filter points within the Z axis range
6:	Build a kd‐tree from the filtered_point
7:	Initialize a graph G with the filtered_points as nodes
8:	**for** each point i in the filtered_points **do**
9:	Find set S of points in kd‐tree within distance >18 from i
10:	**for** each point j in S **do**
11:	Add edge between i and j in G
12:	**end for**
13:	**end for**
14:	Compute maximal cliques in G using the Bron–Kerbosch algorithm
15:	**for** each clique C in the computed cliques **do**
16:	**if** size of C is 3 and angles in △ijk ≥30° for i, j, k in C **then**
17:	Append C to res
18:	**end if**
19:	**end for**
20:	**return** res

#### 2.2.4 Fiducial marker placement planning algorithm

In our procedure, we initially employed a brute‐force algorithm that involved systematically iterating and selecting points that adhered to the aforementioned physical constraints. This approach bore a time complexity of $O(n^{3})$. Given the sheer volume of candidate points, the procedure is far from efficient in identifying fiducial markers.

To improve the efficiency of our algorithm in identifying fiducial marker locations that conform to clinical standards, we employed a kd‐tree as the spatial data structure. Recognized as a partitioning tool for multidimensional space, kd‐trees are well known for their remarkable querying capabilities. This facilitated the implementation of efficient spatial partitioning and querying of the point set. Consequently, the time complexity of the proximity query decreased significantly from the brute‐force $O(n^{2})$. Building on this, we reformulated the problem graphically. Whenever the distance between two points was >18 mm, an edge was formed in the graph. Resorting to graph theory, our challenge evolved into seeking cliques of size 3 in the graph. To address this, we employed the Bron–Kerbosch algorithm, which is designed explicitly to identify all maximal cliques in a graph. Moreover, to align with practical application demands, we incorporated physiological constraints to ensure that all prospective fiducial markers remained proximate to the human body surface. This guaranteed the viability of the fiducial markers at both physical and physiological levels.

Furthermore, we conducted an in‐depth analysis of time complexity. The construction of the kd‐tree has a time complexity of O(mlogm), where *m* represents the number of points after Z axis filtering. Notably, although the proximity query of the kd‐tree may reach O(m) in the worst‐case scenario, its average case complexity typically stands at O(logm). Concerning graph formation, considering the necessity of querying each point and establishing connections between point pairs that meet the criteria, the time complexity for this step is approximately $O(m^{2})$. Finally, regarding maximal clique searching, although the Bron–Kerbosch algorithm grapples with an NP‐hard problem and might, in its extremities, be exponential, it usually outperforms the worst‐case scenario in many real‐world applications, particularly when a graph exhibits certain structures. For a conservative estimate, the complexity can be estimated as $O(3^{\frac{n}{3}})$, where *n* is the vertex count of the graph. Merging the aforementioned analyses, the overall time complexity of the optimized algorithm is $O(mlogm+m^{2}+3^{\frac{n}{3}})$. This represents a significant improvement over the initial $O(n^{3})$ algorithm. The results demonstrated that, in comparison with the original approach, our methodology drastically reduced computational duration, reducing hour‐long processes to mere minutes, while enhancing the precision and utility of the identified points. Algorithm 1 presents the structure of the proposed algorithm.

For fiducial markers that adhere to the aforementioned constraints, their DRRs must be acquired at oblique angles of 45° relative to the X axis to ensure that the fiducial markers do not overlap in the DRRs. If overlapping fiducial markers were discerned within the DRRs, a second set of fiducial markers derived from our algorithm was selected for renewed DRR image generation and examination.

After the aforementioned steps, we filtered out the sets of fiducial markers that met all the constraint conditions. These sets were based on rigorous algorithm models and provided a reliable empirical foundation for this study.

Currently, the location of the fiducial marker relies primarily on the judgment of physicians based on their clinical experience, a process that is not only time‐consuming but also a high barrier to entry. Traditional location planning algorithms using computer programs, such as exhaustive search and iterative screening, face high time complexity because of the large number of computational combinations. This exponential time complexity is unacceptable in practical applications, particularly when the number of candidate points is large. Compared with these traditional methods, our optimization algorithm has significantly reduced time complexity, which can effectively shorten the time of gold‐standard location planning, enhance clinical operability, and improve the efficiency of the overall treatment.

## 3 RESULTS

### 3.1 Experimental data

The experimental data for this research were derived from 3D CT scans of various body parts provided by the hospital, containing structural information of multiple important organs. Fiducial markers do not require internal implantation and can be directly placed on the skin surface. Even if implantation is necessary, these paths are unlikely to necessitate traversal through critical organs. The placement of fiducial markers only needs to meet the geometric constraints required for tracking. Therefore, we selected CT data of patients whose tumors were close to the body surface.

### 3.2 Algorithm validation for superficial tumors with varied placements

**FIGURE 4:**
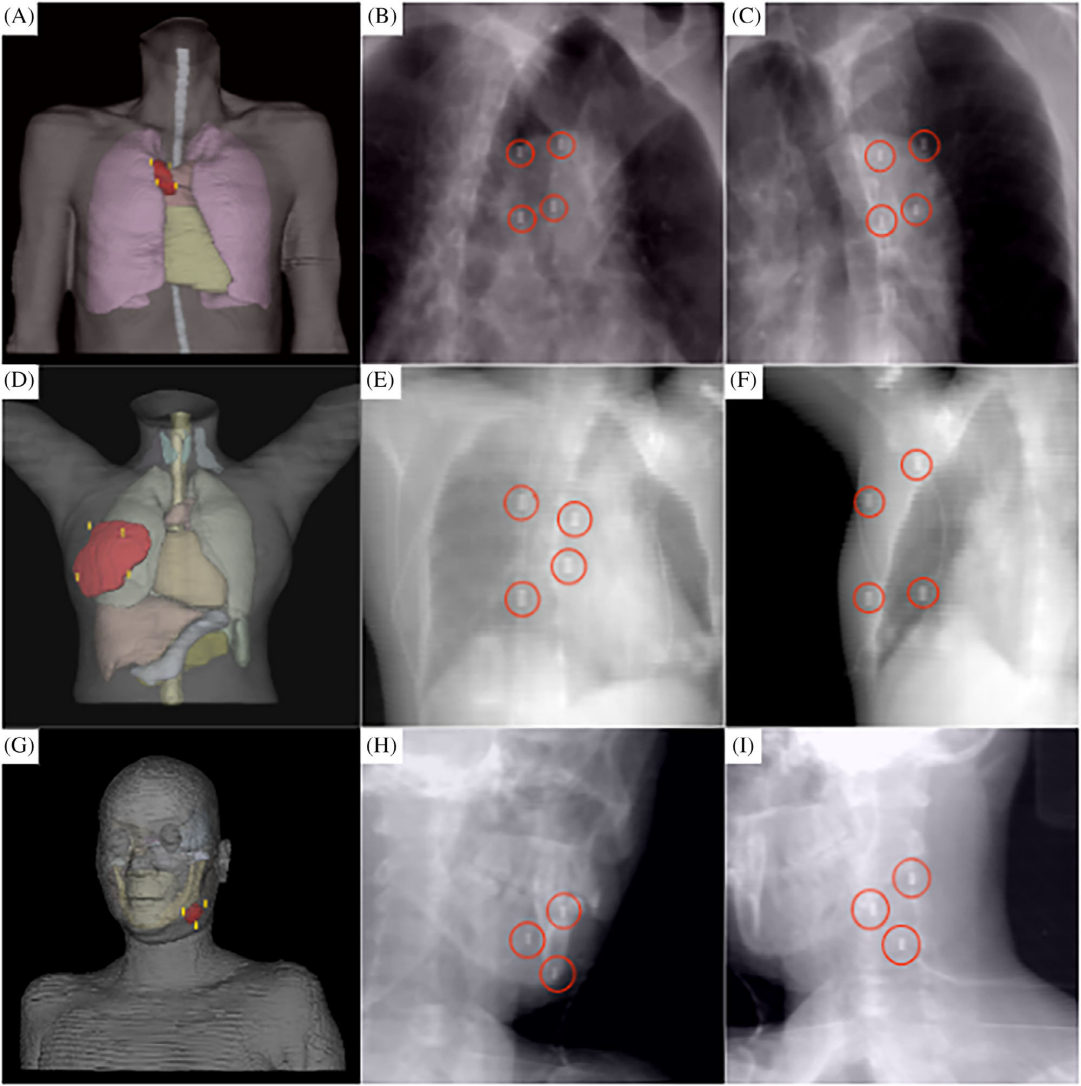
Fiducial marker placement constraint results for different treatment sites.

To validate the stability and reliability of the proposed algorithm, we conducted a retrospective analysis using imaging data from patients who completed the treatment and established three digital human models. Using the proposed algorithm, the locations of the fiducial markers for each representative model were planned. In Figure 4, the first row presents a model showing a tumor located on the chest wall, the second row depicts a tumor within the breast, and the third row shows a tumor in the submandibular gland. For each patient model, the image in the first column displays the visualization of the fiducial markers within the digital human body model, whereas the images in the second and third columns represent the DRRs of the model from a 45° left and right perspectives, respectively. The ideal set of fiducial marker points have been specifically highlighted. Table 1 lists the scoring criteria for the fiducial marker constraints. We specifically highlighted the ideal set of fiducial marker points. Table 1 illustrates the scoring criteria for the fiducial marker constraints, and Table 2 presents the scoring results for the CT images of the three patients. When the proposed algorithm was utilized, the locations of the fiducial marker points strictly adhered to the relevant standards of medical diagnosis and treatment. These algorithmically selected fiducial markers are not only of a suitable number but also offer various viable options for physicians. This advantage is particularly significant in actual clinical operations as it provides greater flexibility for physicians when placing fiducial markers and enables more personalized treatment planning based on individual patient differences. The scoring system employed was based on clinical needs and reflected the specific challenges associated with fiducial marker placement for superficial tumors. Given that the three patients included in our study were relatively straightforward, all achieved full scores. However, we acknowledge that, in clinical practice, especially novice practitioners and physicists who are just beginning to work with the CyberKnife system may face challenges due to a lack of familiarity with the fiducial marker tracking algorithm and spatial visualization skills. This can lead to suboptimal marker placement, resulting in some markers being discarded, or requiring repeated CT localization scans.

**TABLE 1:** Fiducial marker constraint scoring.

Criteria index	Question	Answer	Points
1	Number of markers <50 mm from the tumor	1	1
		2	2
		≥3	3
2	Is the angle between markers >30°?	Yes	2
		No	0
3	Is the distance between markers >18 mm?	Yes	2
		No	0
4	Number of nonoverlapping markers in DRR	1	1
		2	2
		≥3	3

**TABLE 2:** Scoring for the patients with tumors in the chest wall, breast, and submandibular gland.

Criteria	Chest wall	Breast	Submandibular gland
Markers <50 mm from the tumor	3	3	3
Angle between markers >30°?	2	2	2
Distance between markers >18 mm?	2	2	2
Nonoverlapping markers in DRR	3	3	3
**Total points**	10	10	10

## 4 DISCUSSION

We present an intelligent planning method for gold fiducial marker implantation based on B‐spline surface modeling. The approach first reconstructs the 3D expansion surface of tumors using clinical imaging data and subsequently constructs a candidate set of implantation points that meet safety distance requirements, through constrained optimization. By integrating kd‐tree spatial indexing with graph theory and the Bron–Kerbosch maximum clique algorithm, the proposed method efficiently searches for optimal implantation combinations under multiple constraints. Compared with traditional methods, such as exhaustive search or manual placement by physicians, our algorithm offers several distinct advantages. First, it significantly improves accuracy by systematically ensuring compliance with clinical constraints (e.g., ≥18‐mm spacing and ≥30° angular separation between markers, and nonoverlapping markers in DRRs), reducing the risk of human error inherent in manual judgment. Second, it enhances clinical operability by automating the planning process, making it accessible to clinicians with varying levels of experience, including novices, who are unfamiliar with the CyberKnife system. This automation reduces the time and expertise required, compared with traditional trial‐and‐error approaches, which often rely heavily on physician experience and require multiple CT scans for repositioning. Third, the adaptability of our method allows it to generate multiple viable marker sets, offering flexibility for personalized treatment planning across diverse patient anatomies, unlike rigid traditional protocols.

Despite these advancements, our study has notable limitations. First, the sample size was relatively small, with data from only three patients analyzed. This limited cohort size restricts the generalizability of our findings. Second, the selected patients exclusively had superficial tumors located 20–50 mm beneath the epidermis, such as those in the chest wall, breast, and submandibular gland. Although the algorithm performed well for these sites, its efficacy for more complex tumor locations, particularly deep‐seated tumors, such as the lungs, liver, pancreas, and prostate, remains untested. The spatial and anatomical constraints of deeper tumors, such as those with proximity to critical vasculature, nerves, or dynamic respiratory motion, may challenge the current framework of the algorithm. For instance, our validation results (Table 2) indicated suboptimal performance for sites such as the submandibular gland, lungs, and pancreas, where anatomical restrictions occasionally prevented full compliance with the spacing and angular constraints. Future research should expand the sample size and incorporate a broader range of tumor depths and locations to rigorously assess and enhance the robustness of the algorithm.

Although the present study achieved certain results and solved some known problems, there is still room for optimization and exploration. Future research can be deepened in the following aspects: (1) The fiducial marker implantation point planning method can be improved. Although the fiducial marker implantation point planning method in this study was optimized, compared with the brute‐force method, to achieve high‐speed automation for the entire process and improve the inference speed while ensuring the correct planning of fiducial marker implantation points, more advanced methods can be considered. (2) The proposed fiducial marker implantation path planning method can be applied to large datasets. A labeled dataset for fiducial marker implantation path planning can be constructed using the optimal and available fiducial marker implantation paths obtained from planning. This dataset can be further used as a training set for a deep‐learning model, and reasonable paths can be inferred through deep‐learning training. (3) Automatic implantation of fiducial markers can be performed using a robotic arm. This study is a part of a project on development of a fiducial marker implantation navigation robot currently being carried out in our laboratory. This study implemented only a core algorithm for the automatic implantation path of the CyberKnife robotic arm. To truly deploy it using a robotic arm, many functions need to be improved, such as algorithms related to robot operation, to achieve fully automated treatment.

## 5 CONCLUSION

We developed a constraint‐based algorithm for precise planning of fiducial marker placement on tumors close to the body surface. Using a digital model of patient's anatomy, we constructed and optimized a B‐spline surface around the tumor, generating numerous sample points. These points were subsequently filtered through a constraint system that considered the number of fiducial markers, their spatial distance, angular separation, and nonoverlap in the 45° DRRs. Empirical tests validated that this algorithm can efficiently plan fiducial marker placements that meet clinical requirements. It can serve as an important auxiliary tool, assisting physicians in fiducial marker placement, providing crucial technical support for robotic puncture technology. However, this study has limitations, such as a small sample size and restricted tumor types. Future studies should aim to enhance the its robustness and applicability of this algorithm.

## AUTHOR CONTRIBUTIONS

**Jing Huang**: Participation in the entire work; drafting of the manuscript, and final approval of the submitted version. **XianLong Xiong**: Participation in the entire work; drafting of the manuscript, and data analysis. **Cheng Chen**: Drafting and final approval of the submitted version. **YuHan Li, RuiJie Wang**: Data analysis. **Zhi Tao Dai**: Participation in the entire work; drafting of the manuscript, and final approval of the submitted version.

## CONFLICT OF INTEREST STATEMENT

The authors declare that the research was conducted in the absence of any commercial or financial relationships that could be construed as a potential conflict of interest.

## ETHICS APPROVAL AND CONSENT TO PARTICIPATE

This study was approved by the institutional review board of National Cancer Center/National Clinical Research Center for Cancer/Cancer Hospital, and Shenzhen Hospital. We confirm that all methods were performed in accordance with relevant guidelines and regulations.

## CONSENT FOR PUBLICATION

Consent for the publication of data was obtained from all patients. All patients included in this study were aged above 18 years.

## Data Availability

The datasets used and/or analyzed during the current study are available from the corresponding author on reasonable request.
